# Hospital Utilization for Patients With Cirrhosis and Severe Ascites in a Model of Outpatient Paracentesis by Interventional Radiology

**DOI:** 10.7759/cureus.51397

**Published:** 2023-12-31

**Authors:** Mustajab Ahmed, Masuma Islam, Lasha Gogokhia, Carolina Borz-Baba, Dorothy Wakefield, Sofia S Jakab

**Affiliations:** 1 Internal Medicine, Saint Mary's Hospital, Waterbury, USA; 2 Gastroenterology and Hepatology, NewYork-Presbyterian Hospital, Weill Cornell Medicine, New York, USA; 3 Statistics, Department of Research, Saint Francis Hospital & Medical Center, Hartford, USA; 4 Gastroenterology and Hepatology, Yale School of Medicine, New Haven, USA

**Keywords:** hospital utilization, decompensated cirrhosis, interventional radiology, ascites, abdominal paracentesis

## Abstract

Background: Paracentesis is currently performed by interventional radiologists (IR) rather than gastroenterologists/hepatologists or internists. In this model of care, there is usually no evaluation of patients’ renal function or adjustment of their medications at the time of paracentesis. The objectives of this study were to analyze hospital utilization and cirrhosis complications within six months of index outpatient paracentesis by IR and to identify potential areas of improvement in care.

Methods: This is a retrospective study of patients with cirrhosis and ascites who underwent outpatient paracentesis by IR between October 15, 2015, and October 15, 2018, at a tertiary academic medical center. We collected demographics, data on cirrhosis etiology/complications, laboratory tests, provider notes, outpatient paracentesis dates, emergency department (ED) visits, hospitalizations, and ICU admissions within the following six months post index paracentesis. Associations between categorical predictors and clinical outcomes were analyzed using the chi-square test. Associations between quantitative predictors and clinical outcomes were analyzed using the Wilcoxon rank sum test.

Results: Our study included 69 unique patients who had at least one outpatient encounter for paracentesis by IR in the study period. Most patients were men (71%), had alcohol-related cirrhosis as primary etiology (53.6%), an average age of 60 years, and an average Model for End-Stage Liver Disease-sodium (MELDNa) score at baseline of 16. Within six months from index paracentesis, 44 patients (64.7%) underwent repeat IR outpatient paracentesis (total 187 paracenteses, 4.25 paracenteses/patient), 43 patients (62.3%) had ER visits (total 118 ER visits, 2.8/patient), 41 patients (59.4%) had hospital admissions (total 88 admissions, 2.2/patient), and 11 patients required ICU admission. Complications of cirrhosis noted during follow-up included hepatic encephalopathy (40.5%), acute kidney injury (38.2%), upper gastrointestinal (UGI) bleeding (16%), and spontaneous bacterial peritonitis (SBP) in 15%. The mortality rate at six months was 20%. On multivariate analysis, the predictive factors for mortality were older age (p = 0.03) and MELDNa score (p = 0.02). Baseline MELDNa was predictive of acute kidney injury (p = 0.02), UGI bleed (p < 0.01), and ICU admission (p < 0.01), but not of SBP, encephalopathy, ED visit, or hospital admissions. Among patients with more than one paracentesis (64%),six patients underwent transjugular portosystemic shunt (TIPS), but there was no documentation of TIPS consideration in 31 patients (70.4%). A total of 20 patients (29%) were waitlisted for liver transplantation.

Conclusion: In this contemporary cohort of patients with cirrhosis undergoing outpatient IR paracentesis, we found a high rate of short-term cirrhosis complications and hospital utilization, while TIPS consideration was very low. Further data are needed to identify specific gaps in care, but IR paracentesis should be integrated within a multidisciplinary management model, with emphasis on early TIPS in eligible patients, as recommended by the current practice guidelines.

## Introduction

Large-volume paracentesis (LVP) is the first-line therapy recommended for patients with large (grade 3) ascites [1]. Radiologists have become the predominant providers, performing 70-80% of all paracenteses [2,3]. There is limited data on clinical outcomes after inpatient paracentesis by radiology [4], but no information on outpatient paracentesis and how it impacts the care of patients with ascites. This is clinically relevant, as this practice model does not allow for gastroenterology/hepatology evaluation or medication adjustment at the time of the interventional radiology (IR) procedure. There are different proposed models [5-7] to enhance the quality of care for patients with ascites, but without considering the logistics of paracentesis by radiology. The objectives of our study were to analyze healthcare utilization and complications within six months of index outpatient IR paracentesis and to identify potential areas of improvement in care.

## Materials and methods

Design

This is a retrospective analysis conducted at Trinity Health of New England (THoNE), a tertiary care center in Connecticut, USA. Adult patients with an International Classification of Diseases (ICD) code diagnosis of cirrhosis and who had undergone outpatient IR LVP for refractory ascites between October 2015 to October 2018 were included. We excluded patients with age less than 18 years, ascites not related to cirrhosis, presence of acute liver injury or acute liver failure, and the need for other procedures in addition to paracentesis. The study was approved by the IRB.

In terms of the IR model for outpatient paracentesis, patients are commonly referred to LVP by a gastroenterologist who orders the frequency of paracentesis and the laboratory data required, including complete blood count, basic metabolic panel, and the international normalized ratio. At every IR encounter, the patient's vitals are recorded, and the provider reviews the prior 30 days of laboratory data and medication list. Based on the information obtained, concerns regarding the procedures are discussed by IR directly with the prescriber. The local IR guidance for LVP stipulates that patients should be evaluated for the risk of spontaneous bacterial peritonitis (SBP). If no clinical signs of SBP are present, then standard LVP is performed with an appropriate albumin infusion of 8 grams of 25%/liter removed. If patients have a history of SBP or if the initial aspirate is cloudy, then a diagnostic paracentesis would be performed before LVP.

Data collection

Index paracentesis was defined as the first LVP for each unique patient during the study period. The study group completed an extensive chart review and subtracted the following data: patient demographics, cause of cirrhosis, age at index paracentesis, laboratory results, specifically prothrombin time (PT), international normalized ratio (INR), creatinine, serum sodium, total bilirubin, serum albumin, and platelets count. We used the laboratory information to calculate their Model for End-Stage Liver Disease-sodium (MELDNa) score. We reviewed the patient encounters over six months post index paracentesis to determine the frequency and cause of emergency room visits, hospitalizations, and admissions to the intensive care unit (ICU). Provider notes were also reviewed for documentation of discussion regarding transjugular intrahepatic portosystemic shunt (TIPS) as a treatment for refractory ascites. The long-term survival analyses were based on data as of April 2022.

Statistical analysis

Descriptive statistics were calculated for all variables. Frequencies and percentages were used for categorical variables. Mean (SD) for age and median (IQR) for volume of index LVP and MELDNa score were calculated. Chi-square analyses examined the associations between categorical predictors and clinical outcomes. The association between MELDNa score and clinical outcomes was analyzed using the Wilcoxon rank sum test. The relationship between healthcare utilization (any vs. none) and patient characteristics was examined using multivariate logistic regression models. All models included covariate age group (<65, 65+), sex, cause (alcohol, hepatitis B/C, other), and MELDNa score. Similarly, Poisson regression models predicted the number of healthcare encounters, with the same patient characteristics as covariates. Kaplan-Meier estimates were used to calculate overall survival and survival stratified by the causes of cirrhosis. Statistical analysis was performed using SAS statistical software version 9.4 (SAS Institute Inc., Cary, NC). All statistical tests were two-sided and p < 0.05 was considered statistically significant.

## Results

Baseline characteristics

A total of 69 patients underwent at least one outpatient IR paracentesis from October 2015 to October 2018. Most were males (n = 49, 71.01%). The average age was 60 years. Alcohol use was the predominant cause of cirrhosis (n = 37, 53.62%). Fifteen patients (21.74%) had hepatitis B and/or C, while 17 (24.64%) had cirrhosis secondary to other causes. The average MELDNa score at the time of index paracentesis was 16 (Table 1).

**Table 1:** Characteristics of the patients included in the study MELDNa: Model for End-Stage Liver Disease-sodium; LVP: large-volume paracentesis.

Age	
Age, mean (SD)	60.47 (11.46)
Age less than 65 years, N (%)	47 (68.12)
Age 65 years or more, N (%)	22 (31.88)
Gender	
Male, N (%)	47 (71.01)
Female, N (%)	20 (28.98)
Cause of cirrhosis	
Alcohol, N (%)	37 (53.62)
Hepatitis B and/or C, N (%)	15 (21.74)
Other, N (%)	17 (24.64)
MELDNa, median (IQR)	16 (12, 22)
Volume of index LVP, median (IQR)	5000 (2800, 6000)

Healthcare utilization and complications

After the index paracentesis, 44 patients (64.7%) underwent repeat IR outpatient paracentesis for a total of 187 procedures. There were 4.25 paracenteses/patient (median = 3), and 25% of patients had six or more paracenteses. Forty-one patients had a total of 88 hospitalizations (median = 2), and of these, almost a third (13/41 = 31.7%) had three or more admissions. Eleven required ICU admission. Of the 43 patients with ER visits (total 118 ER visits, 2.8/patient), 37% (16/43) had three or more ER visits. The most common reasons for admission were hepatic encephalopathy (HE) and acute kidney injury (AKI). Almost 40% of patients developed at least one episode of HE or AKI. Approximately 15% of patients developed at least one episode of SBP or had an upper gastrointestinal bleed (Table 2).

**Table 2:** Healthcare utilization and complications over six months after index paracentesis

Utilization	N (%)
≥1 Emergency department visit	43 (62.32)
≥1 Hospital admission	41 (59.42)
≥1 Intensive care unit admission	11 (15.94)
≥1 Repeat interventional radiology outpatient paracentesis	44 (63.77)
The most common reasons for admissions	
Hepatic encephalopathy (HE)	28 (40.58)
Acute kidney Injury (AKI)	26 (38.24)
Upper gastrointestinal bleed (UGI bleed)	11 (15.94)
Spontaneous bacterial peritonitis (SBP)	10 (14.49)
Mortality	
Expired within six months of index paracentesis, n (%)	14 (20.3)

Since many patients had multiple hospitalizations and ER visits, they may have had these complications more than once. We examined the relationship between the patient's characteristics (age, sex, cirrhosis cause, and baseline MELDNa score) to complications and six-month mortality. HE and SBP were unrelated to any patient characteristics (data not shown). In contrast, AKI, upper gastrointestinal (UGI) bleeding, and death within six months were all associated with higher MELDNa scores. Patients aged 65 years and older were also more likely to die within six months (Table 3). Sex and cause of cirrhosis were not related to these complications. A total of 20 patients (29%) were waitlisted for liver transplantation.

**Table 3:** Relationship between complications and age, gender, cause of cirrhosis, and baseline MELDNa MELDNa: Model for End-Stage Liver Disease-sodium; AKI: acute kidney injury; UGI: upper gastrointestinal.

Variable	AKI			UGI bleed			Expired within 6 months		
	Yes	No	P-value	Yes	No	P-value	Yes	No	P-value
Age group									
<65, N (%)	15 (57.7)	31 (73.8)	0.17	7 (63.6)	40 (69.0)	0.73	6 (42.9)	41 (74.6)	0.02 (<0.05)
65+, N (%)	11 (42.3)	11 (26.2)		4 (36.4)	18 (31.0)		8 (57.1)	14 (25.4)	
Gender									
Male, N (%)	17 (65.3)	32 (76.1)	0.33	7 (63.6)	42 (72.4)	0.55	9 (64)	40 (72.7)	0.53
Female, N (%)	9 (34.6)	10 (23.8)		4 (36.3)	16 (27.5)		5 (35.7)	15 (27.2)	
Cause of cirrhosis									
Alcohol, N (%)	14 (53.8)	23 (54.7)	0.57	5 (45.5)	32 (55.1)	0.82	8 (57)	29 (52.7)	0.26
Hepatitis B and/or C, N (%)	4 (15.4)	10 (23.8)		3 (27.3)	12 (20.7)		1 (7)	14 (25.4)	
Other, N (%)	8 (30.7)	9 (21.4)		3 (27.3)	14 (24.1)		5 (35.7)	12 (21.8)	
Baseline MELDNa, median (IQR)	21 (15,24)	15 (12,19)	0.02 (<0.05)	23 (16,28)	15 (12,21)	0.009 (<0.05)	23 (15,26)	15 (12,21.5)	0.04 (<0.05)

Consideration of TIPS

TIPS was performed on six patients. There was no documentation of TIPS consideration in 31 (70.4%) of 44 patients who required at least two LVPs within six months. Among those 44 patients, 11 were older than 65 years, possibly the reason for not addressing TIPS eligibility. Baseline MELDNa < 18, which is the recommended limit for elective TIPS for ascites, was present in 20 patients of age 65 or less, but this is an imperfect variable to assess overall TIPS eligibility because it changes over time. Ultimately, TIPS was performed after an average of 389 days from index paracentesis (range = 10-1033 days), and among those patients, three had a baseline MELDNa of more than 20.

Survival

Fourteen (20.29%) patients died within six months of the index paracentesis and another 23 (33.3%) by the end of follow-up (data collected as of April 2022, follow up at least 42 months, average 39 months).

**Figure 1:**
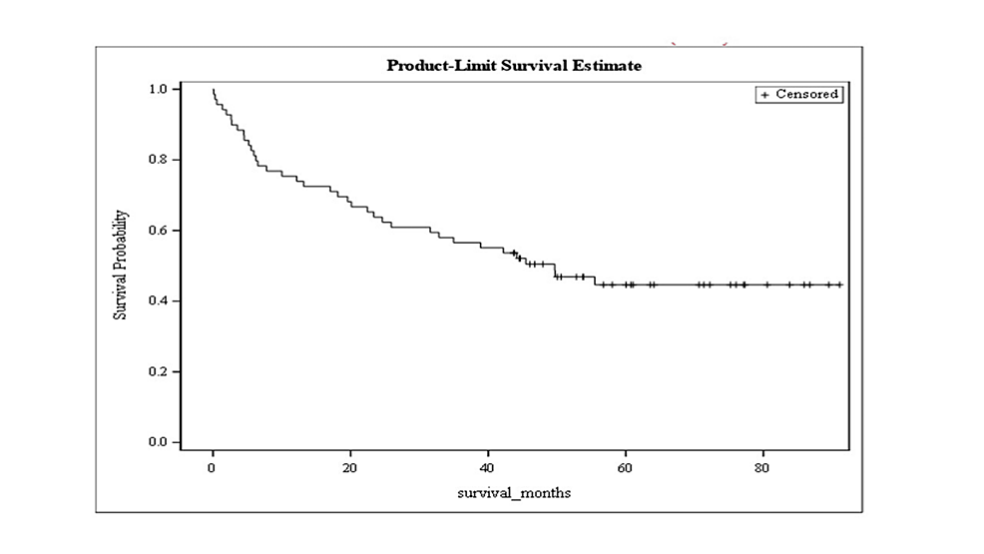
Kaplan-Meier curve representing the overall survival

**Figure 2:**
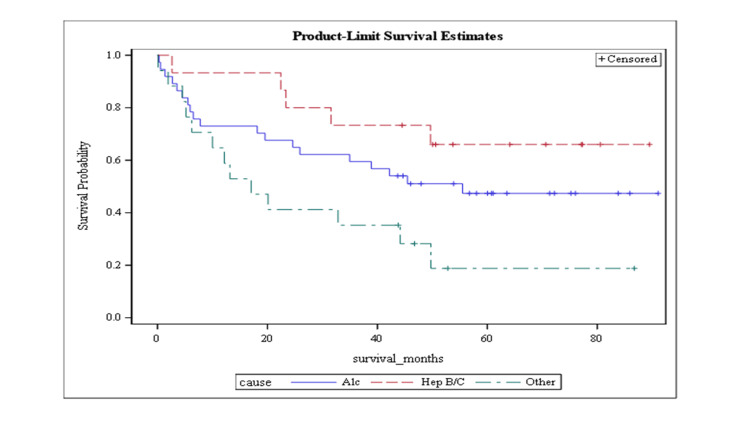
Kaplan-Meier curve representing the product limit survival stratified by the cause of cirrhosis

The mean overall survival was 35.6 months (SE = 2.7) (Figure 1). Figure 2 demonstrates mean survival stratified by cause. Mean survival was 36.3 months (SE = 3.8) for patients with cirrhosis due to alcohol use, 41.8 months (SE = 4.1) for cirrhosis due to hepatitis B or C, and 24.4 months (SE = 4.9) in other etiologies (this category included non-alcoholic steatohepatitis (NASH) but excluded hepatitis B and C), as shown in Figure 2.

Multivariate analyses

Multivariate logistic regression models examined the relationship between patient characteristics (Table 4) and healthcare utilization and six-month mortality (Table 4). Healthcare utilization and death within six months were not related to the cause of cirrhosis. Patients of older age (more than 65 years) were more likely to have a hospital admission and die within six months. Patients with higher baseline MELDNa scores were more likely to have an ICU admission and to die within six months. Poisson regression models predicting hospital utilization (total number of ER visits, hospitalizations, ICU admissions, and repeat IR outpatient paracentesis encounters) demonstrated that patients with higher baseline MELDNa scores had more ICU admissions.

**Table 4:** Results from multivariate regression models MELDNa: Model for End-Stage Liver Disease-sodium.

	≥1 Hospital admission		≥1 ICU admission		Expired within 6 months	
Covariates	OR (95% CI)	P-value	OR (95% CI)	P-value	OR (95% CI)	P-value
Age, <65 vs. 65+	0.10 (0.02, 0.64)	0.01 (<0.05)	0.27 (0.04, 1.83)	0.18 (>0.05)	0.15 (0.03, 0.81)	0.03 (<0.05)
MELDNa score	1 (0.92, 1.08)	0.9 (> 0.05)	1.19 (1.05, 1.34)	0.01 (<0.05)	1.15 (1.03, 1.29)	0.02 (<0.05)

## Discussion

Healthcare utilization

Patients with decompensated cirrhosis require frequent hospitalizations, with high re-admission rates of 26% at 30 days, and 21-71% at three months [8-11]. In patients with ascites, the 30-day readmission rate was reported as 33% [9]. Inpatient IR-guided paracentesis was associated with an increased length of stay, ICU transfer rate, and platelet and fresh frozen plasma transfusions [4], but data on healthcare utilization post outpatient IR-guided procedure are lacking.

The rate of hospitalization in our cohort was 59.42% over six months. The leading reasons for hospitalization in our patients were HE and AKI. A prospective study identified post-LVP AKI in 10.9% of patients [12]. Prior renal impairment and the volume of fluid removed at paracentesis were significant predictors. The risk of AKI increased by 1.24 times per one liter of fluid removal [12]. In our study, the average volume of index LVP was 5 L and the average baseline creatinine was 1.4 (range: 0.4-6.5, median: 1.05), but it is difficult to ascertain if the higher incidence of AKI in our patients was due to lack of adjusting the paracentesis volume or albumin infusion in patients with higher creatinine. In the current model, the frequency of paracentesis is determined by the prescriber. It is unclear how the frequency of treatment changes based on the gastroenterologists’ recommendations, the laboratory data, or patients’ compliance. The medication list and laboratory data are reviewed before the IR encounter, but detailed documentation about the conversation with the prescriber is not always clear. Due to the high incidence of AKI, patients who undergo LVP in IR would benefit from specific protocols to adjust the frequency of procedures, and laboratory evaluation in a patient-centered multidisciplinary communication between the gastroenterologist/hepatologist and IR.

SBP was a reason for hospitalization in 15% of our patients over six months from index outpatient IR paracentesis. Infections are a common reason for readmissions in patients with cirrhosis [11,13]. SBP was reported in only 4% of patients undergoing outpatient paracentesis [14,15] but it is unlikely that the higher rate of SBP in our patient cohort was related to the exposure to IR paracentesis, but rather a reflection of the severity of their liver disease given average MELDNa of 16 and high rate of ICU utilization.

Our study also demonstrated that almost two-thirds of patients required ER evaluation within six months, with a total of 118 ER visits (2.8 per patient). We did not adjust the results for the "weekend effect," but the incidence of ED visits for unplanned LVP was 18.6% in patients who had > four visits during the study period. Our results confirmed data from the literature that the need for urgent LVP is one of the most common reasons for preventable hospital utilization [5] and further process adjustment is necessary to be included when developing a plan to prevent hospital utilization in patients with LVP.

In our study, 16% of patients required ICU care, and higher baseline MELDNa correlated with the need for ICU. We did not find age to be a significant predictor, in contrast to other reports [16]. It is unclear if LVP by IR increases the risk of ICU admission, but it is certain that more active surveillance of this vulnerable group of patients is beneficial.

Survival analysis

The three-year mortality rate in our study was 39.1%, with a mean survival of 35.6 +/- 2.6 months. This is similar to prior reports [17-19]. Mean survival stratified by cause was 36.3 +/- 3.8 months for patients with alcohol-related cirrhosis, 41.8 +/- 4.1 months for cirrhosis due to hepatitis B and C, and 24.4 +/- 4.9 months in other etiologies (that included NASH but excluded hepatitis B and C). As mentioned in other studies [18], the mortality in cirrhosis was highest in patients with NASH. The mortality rate in our cohort was higher in patients with alcohol-related liver disease compared to cirrhosis secondary to viral hepatitis, contrasting with other reports in the literature [18]. We think that this discrepancy is due to the smaller number of patients with viral hepatitis in our study, which reflects the impact of antiviral therapy on the natural history of cirrhosis for a contemporary cohort (2015-2022). The survival analysis determines that NASH continues to represent an important factor for poor prognosis in patients with refractory ascites and emphasizes the need for emerging developments to decrease diabetes mellitus and obesity-related liver disease.

Multivariate analysis

The multivariate analysis examined the predictors of six-month mortality in our cohort. The Cox regression demonstrated that both age ≥ 65 years and higher Model for End-Stage Liver Disease (MELD) scores were related to a shorter survival time. Our results are consistent with known data that mortality is highest in elderly patients with decompensated cirrhosis [16] and that the MELD score is a prognosticator of mortality, length of hospitalization, and disease burden [20].

Reporting survival data for our study does not imply that we consider IR paracentesis a direct cause of mortality, but rather to be interpreted in the larger context of the complex care required by patients with refractory ascites. While the natural progression of cirrhosis was likely the primary driver of complications and mortality in our patients, this is a vulnerable population and significant consequences may result from minor oversights. When paracentesis is performed by IR providers rather than gastroenterologists, the patients may value those appointments for procedures rather than office visits, and they will be seen less frequently for medication adjustment. In addition, a busy radiologist meeting the patient at the time of paracentesis may not prioritize a detailed review of laboratory data or clinical changes, may not be able to reach the gastroenterologist on short notice, and may not limit the volume of paracentesis, leading to complications and higher mortality, which could be avoided. As this model of care is now widespread in the United States, gastroenterologists must be diligent in setting appropriate coordination of procedures and assessment of vital signs, laboratory results, and medications for these patients. The resources and clinical pathways are different across healthcare systems, but gastroenterology and IR need to collaborate to develop protocols to specifically delineate the frequency of labs, provider responsible for timely review, clinical parameters that require a change in ascites volume removed or notification of gastroenterologist, and clinic follow up or asynchronous care involving nurses or advanced practice providers.

Consideration of TIPS

Our study also evaluated the frequency of TIPS consideration in patients with refractory ascites. Practice guidelines recommend consideration for TIPS in all patients with refractory ascites and even for patients with recurrent ascites [1]. Among our patients with more than one paracentesis, only six (10%) underwent TIPS within six months, and there was no documentation of TIPS consideration in 31 patients (70.4%) despite TIPS being available through the same IR department performing LVP. There is a scarcity of literature addressing the causes of underutilization of TIPS in patients with refractory ascites, but potential barriers are concerns regarding post-TIPS complications such as encephalopathy and liver failure [21]. Post-TIPS complications and mortality are increased in the elderly with cirrhosis [22]. The average age of the patients included in our study was 60 years (with 32% of patients above the age of 65 years). Hepatic encephalopathy was very frequent (40.5%) in our cohort. These findings could partially explain the lower candidacy for TIPS, but a systematic early evaluation with adequate documentation of a patient-centered multidisciplinary discussion would be beneficial.

Patients with refractory ascites are referred for transplant evaluation. In our study, 29% were listed as potential candidates. Psychosocial barriers and coexisting comorbidities commonly prevent patients from undergoing a liver transplant, and as such we need alternate safe pathways to care for these patients.

Limitations

Our study is based on retrospective analyses, and the relatively small sample size might decrease the statistical power to define differences among patient groups, especially regarding cirrhosis etiology. While we performed a chart review for short-term outcomes (six months follow-up), we could not control for other variables that could have impacted longer-term outcomes (three-year survival). As we counted every complication encountered by each patient as that mimics the real-life progression of the disease, this type of analysis may lead to an underestimation of the overall utilization or complications.

## Conclusions

Our study analyzes a contemporary cohort of patients with cirrhosis and severe ascites, requiring outpatient IR paracentesis. Over six months from index paracentesis, we found a high rate of hospital utilization (ER visits, hospitalizations, ICU care) and mortality. For most patients, TIPS referral was not considered. Our results emphasize the need to develop specific strategies in the care of those patients to improve their outcomes.

Given the economics of paracentesis reimbursement, radiologists will continue to be the main providers to perform outpatient paracentesis. Clinicians and healthcare systems should be aware of the necessity to closely monitor those patients to prevent admissions for AKI and HE, especially in elderly or patients with high MELD scores, who are at higher risk of dying or requiring ICU transfer. Future practice models to improve the quality of care for patients with ascites should address the optimal integration of IR paracentesis as part of a multidisciplinary management program, as well as appropriate consideration of TIPS.
